# Supplementary material for: Discovery and application of insertion-deletion (INDEL) polymorphisms for QTL mapping of early life-history traits in Atlantic salmon
Source: BMC Genomics. 2010 Mar 8;11:156. doi: 10.1186/1471-2164-11-156 (PMC2838853; doi:10.1186/1471-2164-11-156)
Supplement: Additional file 2 — Information on developed 76 locus single-run INDEL panel in Atlantic salmon. Information on fluorescence labeling, primer concentrations, PCR pooling and links to alignments, INDEL motifs and GENESCAN (Burge and Karlin 1997) predictions of genes/exons are available in html format. [file 1471-2164-11-156-S2.ZIP › Additionalfile2/table17571.html]

Contig Alignment

```
Base	A B C D E F   
1	A A A A A .    
2	A A A A A .    
3	T T T T T .    
4	T T T T T .    
5	C C C C C .    
6	A A A A A .    
7	A A A A A .    
8	T T T T T .    
9	A A A A A .    
10	G G G G G .    
11	T T T T T .    
12	C C C C C .    
13	T T T T T .    
14	T T T T T .    
15	T T T T T .    
16	A A A A A .    
17	T T T T T .    
18	T T T T T .    
19	G G G G G G    
20	A A A A A A    
21	A A A A A A    
22	T T T T T T    
23	A A A A A A    
24	A A A A A A    
25	A A A A A A    
26	G G G G G G    
27	C C C C C C    
28	A A A A A A    
29	G G G G G G    
30	T T T T T T    
31	A A A A A A    
32	C C C C C C    
33	A A A A A A    
34	T T T T T T    
35	T T T T T T    
36	T T T T T T    
37	T T T T T T    
38	A A A A A A    
39	C C C C C C    
40	A A A A A A    
41	A A A A A A    
42	A A A A A A    
43	T T T T T T    
44	A A A A A A    
45	T T T T T T    
46	T T T T T T    
47	T T T T T T    
48	C C C C C C    
49	A A A A A A    
50	C C C C C C    
51	A A A A A A    
52	G G G G G G    
53	T T T T T T    
54	A A A A A A    
55	C C C C C C    
56	A A A A A A    
57	T T T T T T    
58	T T T T T T    
59	T T T T T T    
60	T T T T T T    
61	T T T T T T    
62	T T T T T T    
63	T T T T T T    
64	T T T T T T    
65	T T T T T T    
66	A A A A A A    
67	T T T T T T    
68	T T T T T T    
69	G G G G G G    
70	T T T T T T    
71	A A A A A A    
72	C C C C C C    
73	A A A A A A    
74	A A A A A A    
75	A A A A A A    
76	A A A A A A    
77	T T T T T T    
78	G G G G G G    
79	T T T T T T    
80	C C C C C C    
81	A A A A A A    
82	T T T T T T    
83	T T T T T T    
84	G G G G G G    
85	T T T T T T    
86	A A A A A A    
87	C C C C C C    
88	C C C C C C    
89	A A A A A A    
90	A A A A A A    
91	A A A A A A    
92	G G G G G G    
93	A A A A A A    
94	A A A A A A    
95	A A A A A A    
96	A A A A A A    
97	A A A A A A    
98	T T T T T T    
99	T T T T T T    
100	A A A A A A    
101	T T T T T T    
102	A A A A A A    
103	T T T T T T    
104	T T T T T T    
105	T T T T T T    
106	C C C C C C    
107	T T T T T T    
108	A A A A A A    
109	A A A A A A    
110	T T T T T T    
111	G G G G G G    
112	A A A A A A    
113	T T T T T T    
114	A A A A A A    
115	T T T T T T    
116	T T T T T T    
117	A A A A A A    
118	G G G G G G    
119	T T T T T T    
120	A A A A A A    
121	G G G G G G    
122	C C C C C C    
123	T T T T T T    
124	T T T T T T    
125	C C C C C C    
126	A A A A A A    
127	T T T T T T    
128	T T T T T T    
129	A A A A A A    
130	T T T T T T    
131	C C C C C C    
132	A A A A A A    
133	G G G G G G    
134	T T T T T T    
135	C C C C C C    
136	T T T T T T    
137	A A A A A A    
138	C C C C C C    
139	A A A A A A    
140	A A A A A A    
141	G G G G G G    
142	G G G G G G    
143	A A A A A A    
144	A A A A A A    
145	A A A A A A    
146	T T T T T T    
147	G G G G G G    
148	A A A A A A    
149	A A A A A A    
150	A A A A A A    
151	A A A A A A    
152	A A A A A A    
153	C C C C C C    
154	A A A A A A    
155	A A A A A A    
156	A A A A A A    
157	G G G G G G    
158	T T T T T T    
159	T T T T T T    
160	G G G G G G    
161	T T T T T T    
162	T T T T T T    
163	T T T T T T    
164	A A A A A A    
165	T T T T T T    
166	T T T T T T    
167	G G G G G G    
168	T T T T T T    
169	G G G G G G    
170	C C C C C C    
171	A A A A A A    
172	A A A A A A    
173	A A A A A A    
174	T T T T T T    
175	T T T T T T    
176	G G G G G G    
177	G G G G G G    
178	A A A A A A    
179	A A A A A A    
180	A A A A A A    
181	A A A A A A    
182	G G G G G G    
183	T T T T T T    
184	A A A A A A    
185	A A A A A A    
186	A A A A A A    
187	T T T T T T    
188	G G G G G G    
189	T T T T T T    
190	A A A A A A    
191	A A A A A A    
192	A A A A A A    
193	T T T T T T    
194	A A A A A A    
195	A A A A A A    
196	G G G G G G    
197	A A A A A A    
198	C C C C C C    
199	A A A A A A    
200	T T T T T T    
201	A A A A A A    
202	G G G G G G    
203	T T T T T T    
204	G G G G G G    
205	T T T T T T    
206	T T T T T T    
207	G G G G G G    
208	A A A A A A    
209	T T T T T T    
210	C C C C C C    
211	G G G G G G    
212	T T T T T T    
213	A A A A A A    
214	T T T T T T    
215	C C C C C C    
216	A A A A A A    
217	A A A A A A    
218	A A A A A A    
219	G G G G G G    
220	G G G G G G    
221	A A A A A A    
222	A A A A A A    
223	A A A A A A    
224	T T T T T T    
225	A A A A A A    
226	C C C C C C    
227	A A A A A A    
228	A A A A A A    
229	C C C C C C    
230	G G G G G G    
231	C C C C C C    
232	T T T T T T    
233	A A A A A A    
234	C C C C C C    
235	A A A A A A    
236	A A A A A A    
237	A A A A A A    
238	A A A A A A    
239	G G G G G G    
240	T T T T T T    
241	A A A A A A    
242	A A A A A A    
243	A A A A A A    
244	G G G G G G    
245	A A A A A A    
246	A A A A A A    
247	A A A A A A    
248	T T T T T T    
249	G G G G G G    
250	T T T T T T    
251	T T T T T T    
252	G G G G G G    
253	A A A A A A    
254	C C C C C C    
255	C C C C C C    
256	C C C C C C    
257	T T T T T T    
258	T T T T T T    
259	A A A A A A    
260	A A A A A A    
261	A A A A A A    
262	A A A A A A    
263	A A A A A A    
264	A A A A A A    
265	A A A A A A    
266	T T T T T T    
267	T T T T T T    
268	C C C C C C    
269	T T T T T T    
270	G G G G G G    
271	A A A A A A    
272	G G G G G G    
273	T T T T T T    
274	A A A A A A    
275	A A A A A A    
276	C C C C C C    
277	T T T T T T    
278	T T T T T T    
279	A A A A A A    
280	T T T T T T    
281	T T T T T T    
282	A A A A A A    
283	T T T T T T    
284	T T T T T T    
285	G G G G G G    
286	A A A A A A    
287	T T T T T T    
288	G G G G G G    
289	T T T T T T    
290	T T T T T T    
291	G G G G G G    
292	T T T T T T    
293	A A A A A A    
294	A A A A A A    
295	C C C C C C    
296	T T T T T T    
297	G G G G G G    
298	C C C C C C    
299	A A A A A A    
300	T T T T T T    
301	C C C C C C    
302	A A A A A A    
303	C C C C C C    
304	G G G G G G    
305	G G G G G G    
306	C C C C C C    
307	A A A A A A    
308	C C C C C C    
309	C C C C C C    
310	G G G G G G    
311	A A A A A A    
312	A A A A A A    
313	T T T T T T    
314	A A A A A A    
315	A A A A A A    
316	A A A A A A    
317	G G G G G G    
318	C C C C C C    
319	C C C C C C    
320	G G G G G G    
321	C C C C C C    
322	A A A A A A    
323	C C C C C C    
324	T T T T T T    
325	T T T T T T    
326	A A A A A A    
327	A A A A A A    
328	C C C C C C    
329	C C C C C C    
330	T T T T T T    
331	T T T T T T    
332	G G G G G G    
333	G G G G G G    
334	A A A A A A    
335	T T T T T T    
336	G G G G G G    
337	A A A A A A    
338	T T T T T T    
339	C C C C C C    
340	A A A A A A    
341	T T T T T T    
342	G G G G G G    
343	G G G G G G    
344	G G G G G G    
345	A A A A A A    
346	T T T T T T    
347	T T T T T T    
348	T T T T T T    
349	T T T T T T    
350	T T T T T T    
351	T T T T T T    
352	A A A A A A    
353	A A A A A A    
354	A A A A A A    
355	T T T T T T    
356	A A A A A A    
357	T T T T T T    
358	A A A A A A    
359	T T T T T T    
360	T T T T T T    
361	T T T T T T    
362	T T T T T T    
363	G G G G G G    
364	T T T T T T    
365	A A A A A A    
366	G G G G G G    
367	T T T T T T    
368	A A A A A A    
369	T T T T T T    
370	T T T T T T    
371	G G G G G G    
372	C C C C C C    
373	C C C C C C    
374	T T T T T T    
375	G G G G G G    
376	C C C C C C    
377	A A A A A A    
378	G G G G G G    
379	G G G G G G    
380	T T T T T T    
381	T T T T T T    
382	T T T T T T    
383	T T T T T T    
384	C C C C C C    
385	C C C C C C    
386	A A A A A A    
387	A A A A A A    
388	G G G G G G    
389	A A A A A A    
390	A A A A A A    
391	A A A A A A    
392	A A A A A A    
393	T T T T T T    
394	A A A A A A    
395	C C C C C C    
396	A A A A A A    
397	A A A A A A    
398	T T T T T T    
399	T T T T T T    
400	C C C C C C    
401	T T T T T T    
402	G G G G G G    
403	A A A A A A    
404	T T T T T T    
405	A A A A A A    
406	G G G G G G    
407	A A A A A A    
408	T T T T T T    
409	T T T T T T    
410	T T T T T T    
411	G G G G G G    
412	T T T T T T    
413	T T T T T T    
414	T T T T T T    
415	A A A A A A    
416	A A A A A A    
417	C C C C C C    
418	A A A A A A    
419	A A A A A A    
420	T T T T T T    
421	T T T T T T    
422	A A A A A A    
423	A A A A A A    
424	C C C C C C    
425	A A A A A A    
426	G G G G G G    
427	A A A A A A    
428	A A A A A A    
429	T T T T T T    
430	A A A A A A    
431	A A A A A A    
432	T T T T T T    
433	T T T T T T    
434	T T T T T T    
435	C C C C C C    
436	C C C C C C    
437	A A A A A A    
438	C C C C C C    
439	A A A A A A    
440	A A A A A A    
441	A A A A A A    
442	C C C C C C    
443	A A A A A A    
444	C C C C C C    
445	A A A A A A    
446	C C C C C C    
447	T T T T T T    
448	T T T T T T    
449	T T T T T T    
450	A A A A A A    
451	A A A A A A    
452	G G G G G G    
453	T T T T T T    
454	T T T T T T    
455	T T T T T T    
456	G G G G G G    
457	A A A A A A    
458	A A A A A A    
459	T T T T T T    
460	G G G G G G    
461	T - - T - T  3	 indel  
462	T - - T - T  3	 indel  
463	G - - G - G  3	 indel  
464	T - - T - T  3	 indel  
465	A - - A - A  3	 indel  
466	A - - A - A  3	 indel  
467	A - - A - A  3	 indel  
468	A - - A - A  3	 indel  
469	A A A A A A    
470	A A A A A A    
471	C C C C C C    
472	A A A A A A    
473	T T T T T T    
474	A A A A A A    
475	A A A A A A    
476	T T T T T T    
477	T T T T T T    
478	G G G G G G    
479	G G G G G G    
480	C C C C C C    
481	C C C C C C    
482	A A A A A A    
483	A A A A A A    
484	A A A A A A    
485	A A A A A A    
486	T T T T T T    
487	A A A A A A    
488	A A A A A A    
489	C C C C C C    
490	A A A A A A    
491	C C C C C C    
492	T T T T T T    
493	C C C C C C    
494	T T T T T T    
495	G G G G G G    
496	T T T T T T    
497	G G G G G G    
498	C C C C C C    
499	T T T T T T    
500	G G G G G G    
501	A A A A A A    
502	G G G G G G    
503	A A A A A A    
504	T T T T T T    
505	T T T T T T    
506	T T T T T T    
507	C C C C C C    
508	A A A A A A    
509	A A A A A A    
510	A A A A A A    
511	G G G G G G    
512	A A A A A A    
513	G G G G G G    
514	T T T T T T    
515	G G G G G G    
516	T T T T T T    
517	C C C C C C    
518	T T T T T T    
519	T T T T T T    
520	C C C C C C    
521	A A A A A A    
522	G G G G G G    
523	C C C C C C    
524	T T T T T T    
525	A A A A A A    
526	T T T T T T    
527	T T T T T T    
528	G G G G G G    
529	G G G G G G    
530	G G G G G G    
531	T T T T T T    
532	G G G G G G    
533	T T T T T T    
534	G G G G G G    
535	C C C C C C    
536	G G G G G G    
537	A A A A A A    
538	T T T T T T    
539	T T T T T T    
540	A A A A A A    
541	G G G G G G    
542	G G G G G G    
543	A A A A A A    
544	A A A A A A    
545	A A A A A A    
546	T T T T T T    
547	G G G G G G    
548	T T T T T T    
549	A A A A A A    
550	C C C C C C    
551	G G G G G G    
552	T T T T T T    
553	G G G G G G    
554	C C C C C C    
555	C C C C C C    
556	C C C C C C    
557	T T T T T T    
558	A A A A A A    
559	C C C C C C    
560	T T T T T T    
561	A A A A A A    
562	C C C C C C    
563	A A A A A A    
564	A A A A A A    
565	G G G G G G    
566	T T T T T T    
567	A A A A A A    
568	C G G C G C  3	 transversion  
569	A A A A A A    
570	A A A A A A    
571	T T T T T T    
572	T T T T T T    
573	T T T T T T    
574	G G G G G G    
575	T T T T T T    
576	G G G G G G    
577	A A A A A A    
578	A A A A A A    
579	G G G G G G    
580	A A A A A A    
581	T T T T T T    
582	G G G G G G    
583	T T T T T T    
584	G G G G G G    
585	G G G G G G    
586	C C C C C C    
587	A A A A A A    
588	A A A A A A    
589	A A A A A A    
590	. A A A A A    
591	. G G G G G    
592	. T T T T T    
593	. G G G G G    
594	. A A A A A    
595	. C C C C C    
596	. G G G G G    
597	. C C C C C    
598	. A A A A A    
599	. C C C C C    
600	. C C C C C    
601	. A A A A A    
602	. A A A A A    
603	. . C C C C    
604	. . G G G G    
605	. . G G G G    
606	. . A A A A    
607	. . G G G G    
608	. . A A A A    
609	. . A A A A    
610	. . T T T T    
611	. . T T T T    
612	. . T T T T    
613	. . T T T T    
614	. . T T T T    
615	. . T T T T    
616	. . T T T T    
617	. . T T T T    
618	. . A A A A    
619	. . C C C C    
620	. . C C C C    
621	. . A A A A    
622	. . A A A A    
623	. . G G G G    
624	. . C C C C    
625	. . A A A A    
626	. . T T T T    
627	. . A A A A    
628	. . A A A A    
629	. . G G G G    
630	. . T T T T    
631	. . T T T T    
632	. . G G G G    
633	. . T T T T    
634	. . G G G G    
635	. . C C C C    
636	. . T T T T    
637	. . T T T T    
638	. . C C C C    
639	. . A C A A    
640	. . C C C C    
641	. . A A A A    
642	. . T T T T    
643	. . A A A A    
644	. . A A A A    
645	. . T T T T    
646	. . T T T T    
647	. . C C C C    
648	. . A A A A    
649	. . C C C C    
650	. . T T T T    
651	. . C C C C    
652	. . A A A A    
653	. . C C C C    
654	. . A A A A    
655	. . C C C C    
656	. . A A A A    
657	. . A A A A    
658	. . T T T T    
659	. . T T T T    
660	. . A A A A    
661	. . A A A A    
662	. . T T T T    
663	. . T T T T    
664	. . T T T T    
665	. . G G G G    
666	. . A A A A    
667	. . C C C C    
668	. . C C C C    
669	. . T T T T    
670	. . T T T T    
671	. . T T T T    
672	. . G G G G    
673	. . A A A A    
674	. . C C C C    
675	. . A A A A    
676	. . C C C C    
677	. . A A A A    
678	. . A A A A    
679	. . A A A A    
680	. . G G G G    
681	. . G G G G    
682	. . G G G G    
683	. . G G G G    
684	. . G G G G    
685	. . T T T T    
686	. . C C C C    
687	. . T T T T    
688	. . T T T T    
689	. . T T T T    
690	. . G G G G    
691	. . A A A A    
692	. . C C C C    
693	. . A A C A    
694	. . . C . C    
695	. . . A . A    
696	. . . T . T    
697	. . . A . A    
698	. . . C . C    
699	. . . T . T    
700	. . . A . A    
701	. . . T . T    
702	. . . T . T    
703	. . . A . A    
704	. . . . . C    
705	. . . . . G    
706	. . . . . A    
707	. . . . . T    
708	. . . . . A    
709	. . . . . A    
710	. . . . . A    
711	. . . . . C    
712	. . . . . A    
713	. . . . . T    
714	. . . . . C    
715	. . . . . A    
716	. . . . . C    
717	. . . . . T    
718	. . . . . G    
719	. . . . . G    
720	. . . . . A    
721	. . . . . A    
722	. . . . . G    
723	. . . . . A    
724	. . . . . C    
725	. . . . . T    
726	. . . . . C    
727	. . . . . A    
728	. . . . . G    
729	. . . . . C    
730	. . . . . C    
731	. . . . . G    
732	. . . . . T    
733	. . . . . G    
734	. . . . . T    
735	. . . . . T    
736	. . . . . G    
737	. . . . . T    
738	. . . . . T    
739	. . . . . T    
740	. . . . . A    
741	. . . . . C    
742	. . . . . A    
743	. . . . . G    
744	. . . . . T    
745	. . . . . C    
746	. . . . . C    
747	. . . . . T    
748	. . . . . A    
749	. . . . . G    
750	. . . . . T    
751	. . . . . A    
752	. . . . . A    
753	. . . . . C    
754	. . . . . A    
755	. . . . . T    
756	. . . . . G    
757	. . . . . T    
758	. . . . . T    
759	. . . . . T    
760	. . . . . G    
761	. . . . . T    
762	. . . . . G    
763	. . . . . C    
764	. . . . . C    
765	. . . . . C    
766	. . . . . T    
767	. . . . . G    
768	. . . . . C    
769	. . . . . G    
770	. . . . . G    
771	. . . . . C    
772	. . . . . A    
773	. . . . . A    
774	. . . . . G    
775	. . . . . C    
776	. . . . . A
```
